# Lithiophilic Reduced Graphene Oxide/Carbonized Zeolite Imidazolate Framework-8 Composite Host for Stable Li Metal Anodes

**DOI:** 10.3390/ma17174300

**Published:** 2024-08-30

**Authors:** Sang-Won Jeong, Byeong Il Oh, Eun Seo Chang, Jeong-Ann Park, Hyun-Kyung Kim

**Affiliations:** 1 Department of Battery Convergence Engineering, Kangwon University, 1, Kangwondaehak-gil, Chuncheon 24341, Republic of Korea; jkw4005@naver.com (S.-W.J.); obi99@naver.com (B.I.O.); aglada@kangwon.ac.kr (E.S.C.); 2 Interdisciplinary Program in Advanced Functional Materials and Devices Development, Kangwon National University, Chuncheon 24341, Republic of Korea; 3 Department of Environmental Engineering, Kangwon National University, Chuncheon 24341, Republic of Korea; 4 Department of Integrated Energy and Infra System, Kangwon National University, Chuncheon 24341, Republic of Korea

**Keywords:** Li metal anode, lithiophilic, reduced graphene oxide/carbonized ZIF-8 composite

## Abstract

Lithium (Li) metal is regarded as a next-generation anode material owing to its high energy density. However, issues such as dendritic growth and volume changes during charging and discharging pose significant challenges for commercialization. We propose using lithiophilic reduced graphene oxide (rGO) and carbonized zeolite imidazolate framework-8 (C-ZIF-8) composites as host materials for Li to address these problems. The rGO/C-ZIF-8 composites are synthesized through a simple redox reaction followed by carbonization and are characterized using X-ray diffraction (XRD), scanning electron microscopy (SEM), and X-ray photoelectron spectroscopy (XPS). The roles of chemical composition, characteristics, and morphology are demonstrated. As a result of these favorable structural and functional properties, the Li symmetric cell with rGO/C-ZIF-8 exhibits a stable voltage profile for more than 100 h at 1 mA cm^−2^ without short-circuiting. A relatively low Li plating/stripping overpotential of ~101.5 mV at a high current density of 10 mA cm^−2^ is confirmed. Moreover, a rGO/C-ZIF-8-Li full cell paired with a LiFePO_4_ cathode demonstrates good cyclability and rate capability.

## 1. Introduction

The requirement for energy storage devices with high energy density has become critical with the rapid increase in demand for electric vehicles. However, the currently commercialized graphite anode, with a theoretical capacity of 372 mAh g^−^^1^, does not satisfy these requirements [1,2]. Lithium (Li) metal has been emphasized as a next-generation anode for Li-S, Li-O_2_, and all solid-state batteries because of its high theoretical capacity (3860 mAh g^−^^1^), low electrochemical reduction potential (−3.04 V vs. SHE), and low gravimetric density (0.534 g cm^−^^3^) [3]. Despite these advantages, Li metal anodes face significant challenges due to Li dendrite growth and substantial volume changes, resulting in the destruction of the solid electrolyte interphase (SEI) layer. This destruction causes the exposed Li surface to react with the electrolyte, resulting in repeated SEI layer regeneration and, consequently, high overpotential and cell short circuits [4]. Thus, Li dendrite growth degrades lifespan characteristics and rate performance, causing safety issues and posing significant challenges for commercialization [5,6].

Despite these challenges, innovative solutions are being developed to overcome the limitations of Li metal anodes. Various approaches have been explored to address the challenges posed by Li dendrite growth [7,8,9], but each has significant limitations that hinder its effectiveness. First, solid-state electrolytes, which are relatively safer than liquid electrolytes, can mechanically suppress dendrite growth. However, they have low ionic conductivity and high interfacial resistance between the active material and electrolyte. These issues significantly impede the overall performance of the battery and present substantial barriers to commercialization [10,11,12]. Second, the introduction of electrolyte additives to form a stable SEI layer can inhibit dendrite growth. However, this solution is not without its flaws. The SEI layer often undergoes repeated breakdown and regeneration during charge/discharge cycles, leading to continuous consumption of the electrolyte and loss of active Li. Additionally, the significant volume changes during cycling exacerbate these issues, ultimately limiting the long-term stability of the battery [13,14,15]. Finally, the introduction of three-dimensional (3D) hosts with large specific surface areas can reduce local current density, thereby inhibiting dendrite growth and providing space to accommodate volume changes. Initially, studies were conducted on 3D metal framework structures such as 3D nickel foam and 3D porous copper current collectors [16,17,18]. However, these structures have reduced energy density owing to their high specific gravity. This trade-off between structural stability and energy density makes it challenging to develop a viable solution for practical applications. Consequently, carbon-based host materials (such as CNT, graphene, and carbon cloth) have been reported as stable hosts for Li metal anodes [19,20,21,22]. However, most carbon-based host materials exhibit lithiophobic characteristics, which hinder uniform Li plating/stripping [23]. Therefore, the introduction of additional lithiophilic materials is essential to achieve stable Li plating/stripping and inhibit dendrite growth.

Recent research regarding the increased adsorption of Li on reduced graphene oxide (rGO) with structural defects, such as oxygen functional groups has been reported [24,25]. In particular, oxygen functional groups on rGO, especially carbonyl groups, act as lithiophilic sites that facilitate homogeneous Li metal deposition and prevent dendrite growth [26,27,28,29]. Moreover, 3D lithiophilic host materials prevent Li dendrite growth [30,31,32]. A zeolite imidazolate framework-8 (ZIF-8) is a 3D structure composed of Zn^2+^ and 2-methylimidazole organic linkers, which possesses a sodalite zeolite-type structure [33,34] providing excellent thermal and chemical stability [35]. Moreover, a 3D framework containing lithiophilic materials such as Zn and N plays a crucial role in improving the uniformity of Li deposition and reducing the Li nucleation energy barrier [31,36,37,38], and it has a large specific surface area and pore volume, which can reduce local current density and accommodate significant volume changes. However, owing to the combination of Zn and N, it exhibits lithiophobic characteristics. Therefore, after carbonization, ZIF-8 can be transformed into porous carbon-containing Zn clusters and N atoms.

Herein, we synthesized a rGO/C-ZIF-8 composite including 3D lithiophilic carbonized ZIF-8 using a simple redox process and carbonization method for Li metal reservoir host materials. Because of the lithiophilic Zn and N of C-ZIF-8 and oxygen functional groups on rGO in the composite, it demonstrates stable plating/stripping behavior in Li symmetric cells; furthermore, it can achieve cyclability and rate capability in a full cell system with a LiFePO_4_ cathode.

## 2. Experimental Methods

### 2.1. Preparation of rGO/C-ZIF-8

rGO/C-ZIF-8 composites were synthesized from commercial graphene oxide (GO, Angstron Materials) and zinc metal (<10 μm, ≥98%, Aldrich) through a spontaneous redox reaction [39], with the addition of 2-methylimidazole (99%, Aldrich) and carbonization. First, 20 g of GO was ultrasonicated in 380 mL of deionized water at 10 °C for 45 min using a 750 W sonicator operating at a frequency of 20 kHz. Subsequently, 200 mg zinc metal powder was added to the GO solution and stirred at room temperature over 12 h. During this process, ZnO nanoparticles were deposited on the GO surface, accompanied by the partial reduction of GO to partially reduced graphene oxide (PrGO) via an electrochemical redox reaction [39]. Next, 24.2 g of 2-methylimidazole was added to the PrGO/ZnO solution and stirred at room temperature for 24 h. Simultaneously, the molar ratio of Zn^2+^–2-methylimidazol–deionized water was set to 1:60:2228 [40]. Subsequently, the obtained composites were freeze-dried at −50 °C for three days. Finally, the obtained powders were carbonized by heating at a rate of 5 °C/min to 600 °C in Ar for 3 h (gas flow rate: 500 cc/min). To synthesize rGO for the control samples, GO was heated at 600 °C in Ar for 3 h.

### 2.2. Materials Characterization

X-ray diffraction (XRD, X’Pert PRO MPD, PANalytical) was used to investigate the structural properties from 5° to 80°, measured using Cu Kα radiation (λ = 1.5419 Å). The morphologies and microstructures of the synthesized composites were examined by field emission scanning electron microscopy (FE-SEM, Hitachi S-4800, Hitachi, Tokyo, Japan). X-ray photon electron spectroscopy (XPS, Al Kα X-ray source (E_p_ = 1486.7 eV) at a pressure of 5 × $10^{-9}$ mbar or better, Thermo VG (ThermoFisher SCIENTIFIC, Waltham, MA, USA)) was performed to obtain the elemental compositions and chemical states of the different carbon materials.

### 2.3. Electrochemical Measurements

The electrochemical properties of rGO/C-ZIF-8 and rGO-based working electrodes were analyzed using a CR2032 coin cell at room temperature. Various electrodes were prepared by mixing 80 wt.% of the active material (rGO/C-ZIF-8, rGO) and 20 wt.% polyvinylidene fluoride (PVDF, Aldrich, St. Louis, MO, USA) as a binder dissolved in *N*-methyl pyrrolidone (NMP). To fabricate the electrodes, the mixed slurry was coated on Cu foil by a doctor blade and dried in an oven at 70 °C for 12 h. Subsequently, working electrodes with an area of ~1.131 cm^2^ were assembled in coin cells with a Li disk (ϕ16(D) × 0.4 mm(T)) as the reference electrode and counter electrode in an Ar-filled glove box (MBRAUN, O_2_, and H_2_O < 0.1 ppm). In addition, 1 M lithium bis(trifluoromethanesulfonyl)imide (LiTFSI) in a mixture of 1,3-dioxolane (DOL) and 2-dimethoxyethane (DME) (DOL/DME, volume ratio 1:1) with 3 wt.% LiNO_3_ was used as the electrolyte, and microporous polyethylene film (Celgard 2400, Charlotte, NC, USA) was used as a separator. The coin cells were assembled in the following order: bottom casing, working electrode, separator, Li disk, spacer, wave spring, and top casing. Galvanostatic charge/discharge tests were conducted on a battery cycler (BTS-4008, NEWARE, Shenzhen, China). To form a stable solid electrolyte interphase (SEI), the batteries were first cycled three times between 0.01 V and 1 V at 50 μA. After pre-cycling, 4 mAh cm^−^^2^ of Li was plated on rGO/C-ZIF-8, rGO, and Cu foil with a current density of 1 mA cm^−^^2^. LiFePO_4_ (LFP) was fabricated as a cathode material for testing the full cell of the lithiated anodes (rGO/C-ZIF-8-Li, rGO-Li, and Cu-Li). The cathode was composed of 89 wt.% LFP, 3 wt.% carbon black conductive material, and 8 wt.% Polyvinylidene fluoride (PVDF) binder dissolved in NMP (N-Methyl-2-Pyrrolidone) (NMP). The loading mass of LFP was approximately 8.9 mg cm^−^^2^, and the density of LFP was approximately 1.7 g cm^−^^3^. The full cells were cycled from 2.5 V to 4.2 V at 1 C.

## 3. Results and Discussion

### 3.1. Preparation and Characterization of rGO/C-ZIF-8

**Figure 1:**
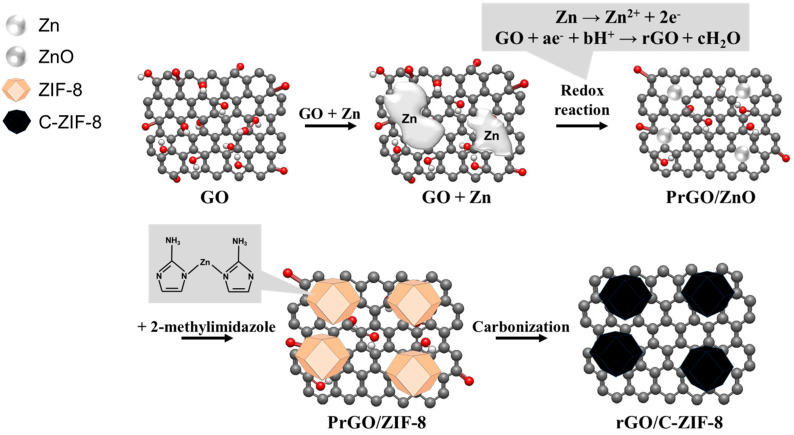
Schematic diagram of the rGO/C-ZIF-8 composite synthesis procedure.

Figure 1 illustrates the synthesis procedure of rGO/C-ZIF-8 composites, which consists of a spontaneous redox reaction, followed by the addition of 2-methylimidazole and carbonization. First, Zn reduces GO partially because of their standard reduction potential difference. The reduction potential of Zn is −0.76 V (Zn/Zn^+^ vs. SHE), and the potential of functional groups on GO is lower (−0.4 V vs. SHE). Hence, the Zn metal particles are transformed to ZnO, and GO undergoes partial spontaneous reduction [39]. Next, 2-methylimidazole is added to the PrGO/ZnO dispersion to induce the formation of ZIF-8 on the graphene [40]. Finally, the PrGO is reduced, and the ZIF-8 is carbonized to synthesize the rGO/C-ZIF-8 composite.

**Figure 2:**
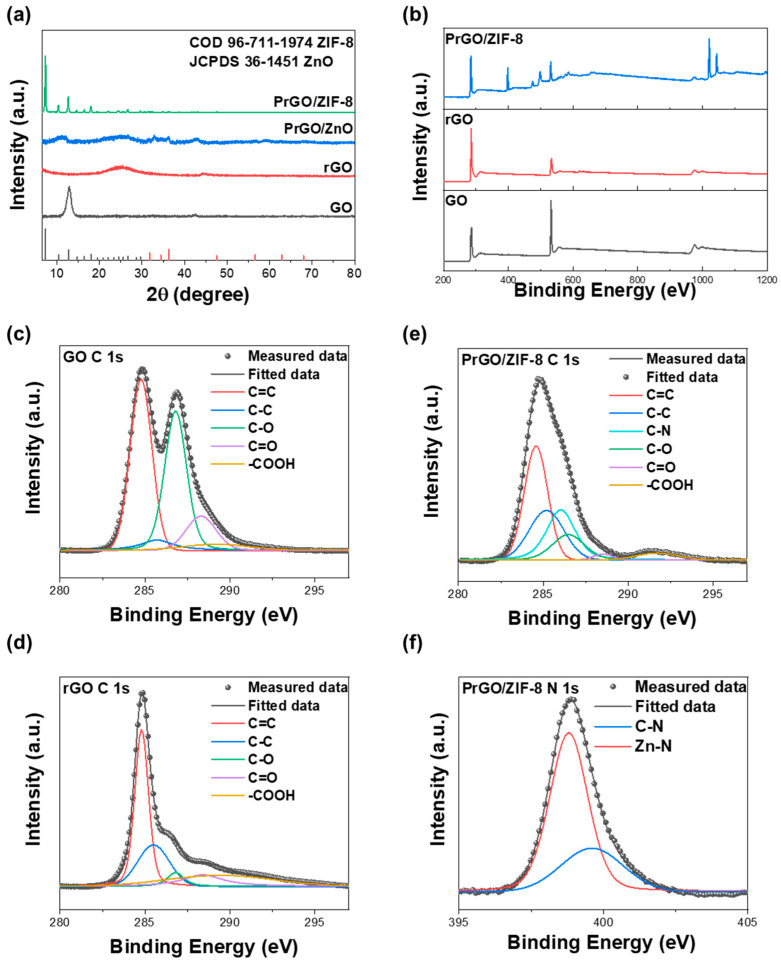
(**a**) XRD patterns, (**b**) XPS survey spectra of GO, rGO, and PrGO/ZIF-8, (**c**–**e**) C 1s spectra of GO, rGO, and PrGO/ZIF-8, and (**f**) N 1s spectra of PrGO/ZIF-8.

The XRD patterns are illustrated in Figure 2a. The absence of the (002) peak of GO in the XRD pattern of rGO indicates thermal reduction of GO to rGO. This sample exhibited the typical (002) plane of rGO at 2 *θ* = ~24° because of the reduction [39]. In the case of PrGO/ZnO, the coexistence of peaks corresponding to GO and rGO confirms that GO was partially reduced, and the presence of characteristic peaks of ZnO (JCPDS card No. 36-1451) indicates that Zn metal was oxidized to ZnO. Furthermore, the presence of peaks of ZIF-8 (COD card No. 96-711-1974) confirms the successful synthesis of PrGO/ZIF-8 through the addition of 2-methylimidazole [40]. Figure 2b illustrates the XPS survey spectra of GO, rGO, and PrGO/ZIF-8. The spectra for GO and rGO indicate the presence of carbon (C 1s) and oxygen (O 1s) at approximately 285 and 533 eV, respectively. However, in the spectra for PrGO/ZIF-8, the presence of ZIF-8 results in additional peaks corresponding to nitrogen (N 1s) and zinc (Zn LMM, Zn 2p_3/2_, and Zn 2p_1/2_) at approximately 398, 498, 1020, and 1043 eV, respectively, together with the peaks for carbon and oxygen [41].

Figure 2c–f illustrate the C 1s and N 1s spectra of each sample. The C 1s spectrum was deconvoluted into components representing the carbon skeleton (C=C, C-C) and oxygen functional groups such as epoxy (C-O), carbonyl (C=O), and carboxyl groups (-COOH) [42]. For PrGO/ZIF-8, the C 1s spectrum was additionally deconvoluted to include C-N bonding, confirming the presence of 2-methylimidazole within the composite. Additionally, the deconvolution of the N 1s spectrum for PrGO/ZIF-8 revealed the presence of C-N and Zn-N bonding, further verifying the incorporation of ZIF-8 within the composite [43].

**Figure 3:**
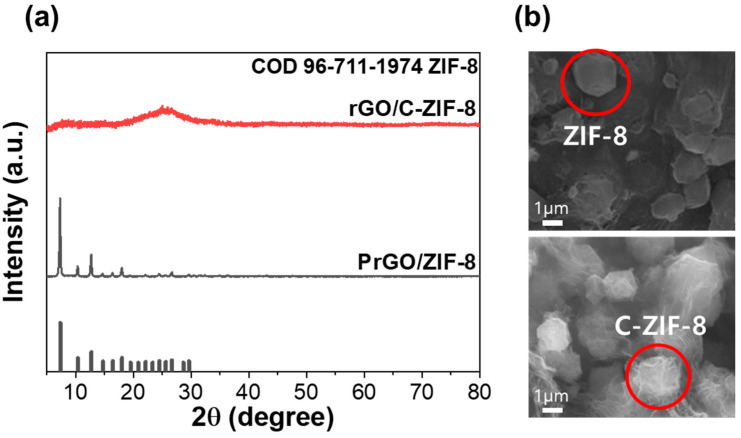
(**a**) XRD patterns and (**b**) SEM images of PrGO/ZIF-8 and rGO/C-ZIF-8.

Figure 3a illustrates the XRD patterns of PrGO/ZIF-8 and rGO/C-ZIF-8 before and after the carbonization process. The XRD pattern of PrGO/ZIF-8 before carbonization exhibits peaks associated with ZIF-8 (COD card No. 96-711-1974). However, after carbonization, the XRD pattern of rGO/C-ZIF-8 shows only peaks related to carbon, indicating successful carbonization [30,31].

Figure 3b illustrates the SEM images of PrGO/ZIF-8 and rGO/C-ZIF-8. The SEM image of PrGO/ZIF-8 before carbonization reveals the presence of 1 μm-sized ZIF-8 on the graphene. The SEM image of rGO/C-ZIF-8 after carbonization indicates that the morphology of ZIF-8 remains intact, confirming structure preservation during the carbonization process [44].

### 3.2. Li Plating Process on the rGO/C-ZIF-8 Composite

**Figure 4:**
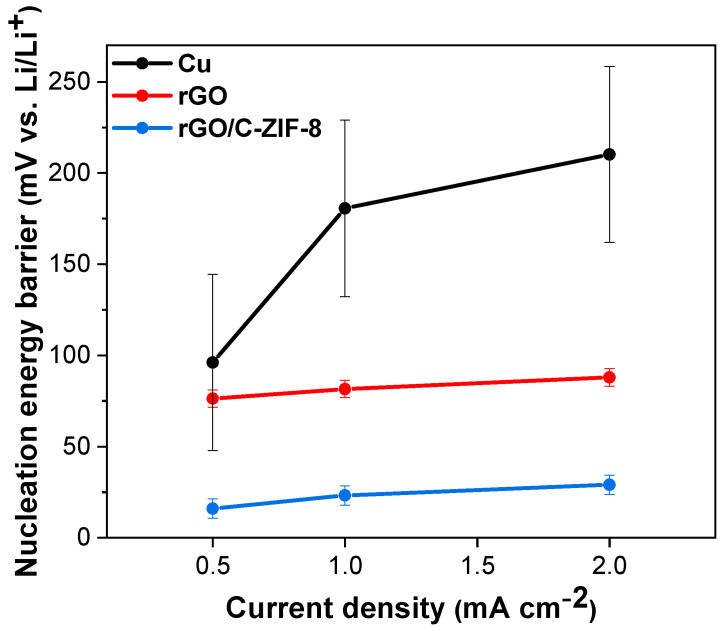
Nucleation energy barrier of Li on Cu-Li, rGO-Li, and rGO/C-ZIF-8-Li at various current densities (0.5, 1, and 2 mA cm^−^^2^) with 1 mAh cm^−^^2^ areal capacity.

To evaluate the electrochemical properties of the prepared rGO/C-ZIF-8 as a Li metal host material, symmetric cell tests were conducted using rGO/C-ZIF-8, Cu, and rGO host electrodes. Before Li plating, the half cells were cycled three times at 0–1 V (vs. Li/Li^+^) at 0.05 mA cm^−^^1^ to form a stable SEI film (Figure S1) [20,45,46]. The Li storage capacity of the rGO/C-ZIF-8 electrode between 0 and 1 V was approximately 2.421 mAh cm^−^^2^, which was superior to the Li storage capacity values of Cu (~0.001 mAh cm^−^^2^) and rGO (~1.096 mAh cm^−^^2^). When Li was plated on each electrode at various current densities with an areal capacity of 1.5 mAh cm^−^^2^ (Figure S2), it was confirmed that the Li plating overpotential of the rGO/C-ZIF-8 electrode was lower than those of the Cu and rGO host electrodes (Figure 4 and Table S1). This result indicates that the Zn and N of C-ZIF-8 and oxygen functional groups on rGO act as lithiophilic sites in the composite to reduce the Li plating overpotential compared with those of rGO and Cu [47]. 

### 3.3. Li Plating/Stripping Behavior of the rGO/C-ZIF-8-Li Electrode

**Figure 5:**
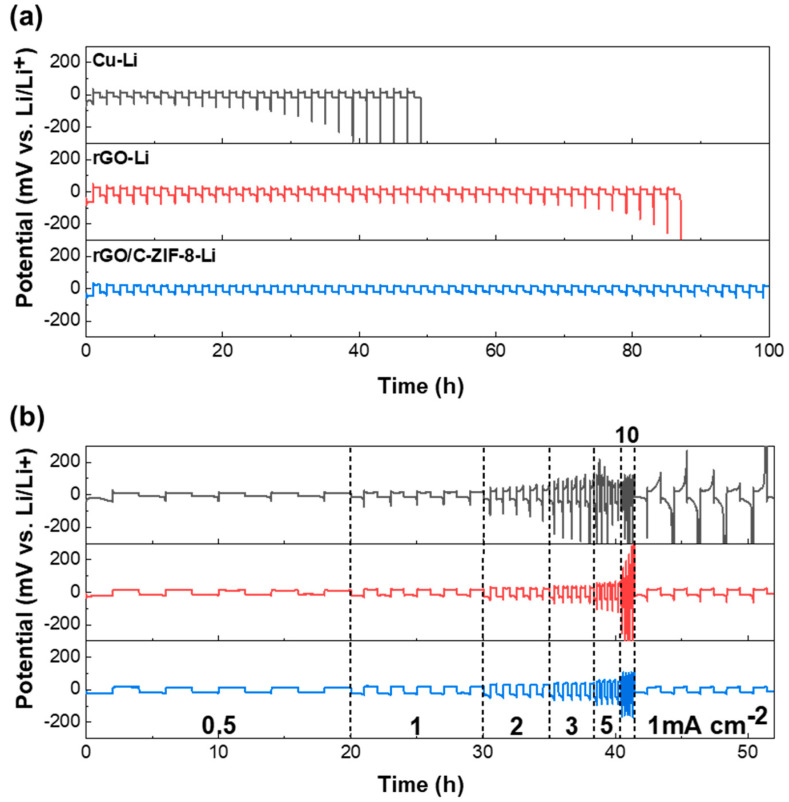
(**a**) Cycling performance of Cu-Li, rGO-Li, and rGO/C-ZIF-8-Li at a current density of 1 mA cm^−^^2^ with an areal capacity of 1 mAh cm^−^^2^. (**b**) Rate performance of Cu-Li, rGO-Li, and rGO/C-ZIF-8-Li at current densities from 0.5 to 10 mA cm^−^^2^ with an areal capacity of 1 mAh cm^−^^2^.

Li-plated samples (Cu-Li, rGO-Li, and rGO/C-ZIF-8-Li) were fabricated at a 1 mA cm^−^^2^ current density for 4 h (areal capacity: 4 mAh cm^−^^2^) for the symmetric cell test. As illustrated in Figure 5a and Table S2, the symmetric cells of the Cu-Li and rGO-Li electrodes failed after 49 and 87 h with high initial plating overpotentials of 66.7 and 38.4 mV, respectively. The rapid increase in voltage hysteresis is attributed to the formation of cracks within the electrode due to volume changes, leading to localized Li-ion flux concentration at the crack sites and subsequent dendritic growth, which accelerates electrolyte depletion. However, rGO/C-ZIF-8-Li demonstrated lower plating overpotential (30.5 mV), and the voltage profile did not show a gradual increase, indicating the absence of a short circuit via dendrite for more than 100 h. rGO/C-ZIF-8 is lithiophilic because of the formation of an N-doped carbon coating, and its porous structure enhances Li ion distribution while also acting as a barrier to prevent Li dendrite formation, leading to improved cycling performance. Consequently, improved cyclic stability and lower overpotential confirmed that using rGO/C-ZIF-8 composites for Li host materials could lead to a stable Li plating/stripping performance. Moreover, Figure 5b and Table S3 show the rate performance of each sample at current densities from 0.5 to 10 mA cm^−^^2^ with an areal capacity of 1 mAh cm^−^^2^. The symmetric cell with Cu-Li showed high plating/stripping overpotential values of 35.0, 51.2, 86.9, 162.2, 370.3, and 224.5 mV, and rGO-Li showed high plating/stripping overpotential values of 21.6, 29.9, 41.2, 59.3, 90.2, and 203.4 mV at current densities of 0.5, 1, 2, 3, 5, and 10 mA cm^−^^2^, respectively. In comparison, the rGO/C-ZIF-8-Li symmetric cell demonstrated pronounced lower overpotential values of 21.2, 27.5, 40.8, 52.1, 72.6, and 123.5 mV at 0.5, 1, 2, 3, 5, and 10 mA cm^−^^2^. 

### 3.4. Practical Applicability of rGO/C-ZIF-8-Li in Full Cells

**Figure 6:**
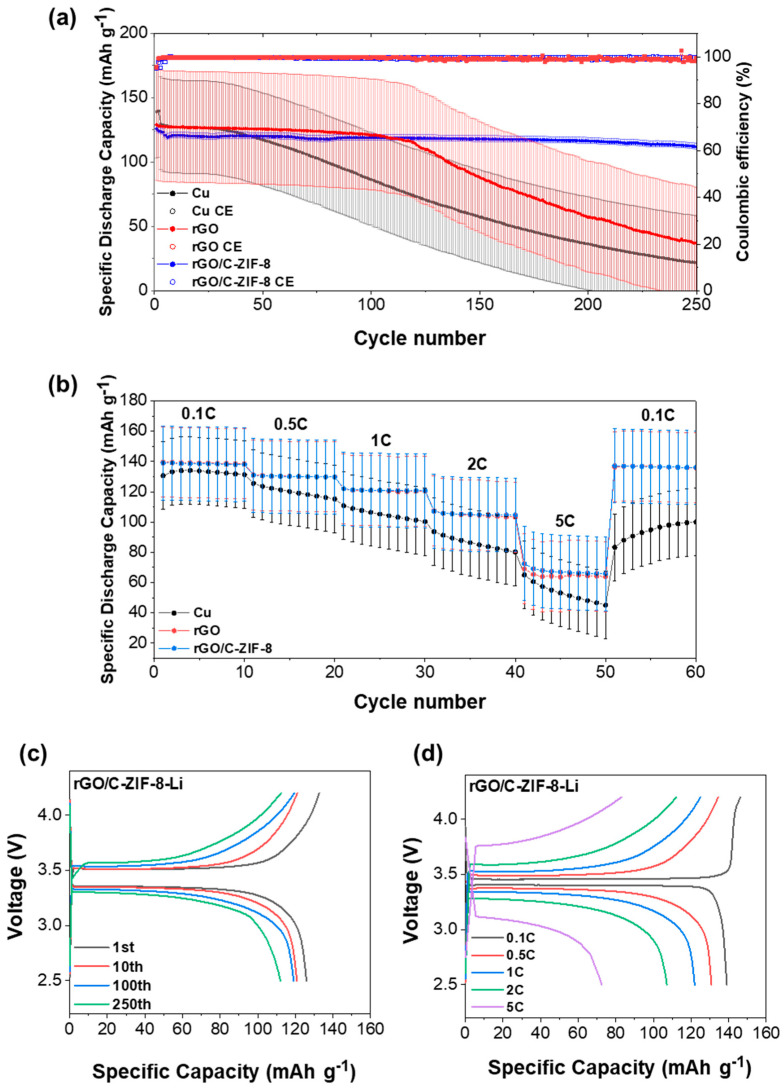
(**a**) Cycling performance of LFP full cells with Cu, rGO, and rGO/C-ZIF-8. (**b**) Rate performance of LFP full cells with Cu, rGO, and rGO/C-ZIF-8. (**c**) Charge/discharge profiles at different cycles at 1 C of rGO/C-ZIF-8-Li. (**d**) Charge/discharge profiles at different rates of rGO/C-ZIF-8-Li.

To evaluate the potential application of the rGO/C-ZIF-8-Li electrode, full cells based on the rGO/C-ZIF-8-Li anode and an LFP(LiFePO_4_) cathode were assembled. As illustrated in Figure 6a,c and Figure S3, the cycling performance of the rGO/C-ZIF-8-Li full cell exhibited desirable cycling stability with a high discharge capacity of 112 mAh g^−^^1^ and Coulombic efficiency (CE) of 99.8% at a 1 C-rate for 250 cycles, compared with rGO-Li (37 mAh g^−^^1^, 98.7%) and Cu-Li (21.9 mAh g^−^^1^, 98.4%). Moreover, the rGO/C-ZIF-8-Li full cell exhibited a high-rate capability of 69.1 mAh g^−^^1^ at a 5 C-rate, compared with Cu-Li (45.1 mAh g^−^^1^) and rGO-Li (63.9 mAh g^−^^1^), as illustrated in Figure 6b,d and Figure S4. 

**Figure 7:**
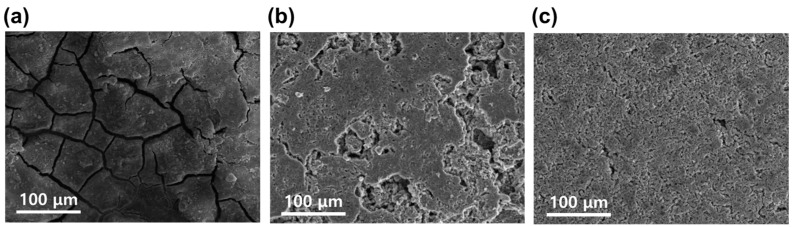
SEM images of the (**a**) Cu-Li, (**b**) rGO-Li, and (**c**) rGO/C-ZIF-8-Li electrodes after 250 cycles in an LFP full cell.

To support electrochemical performance, the images of the Cu-Li, rGO-Li, and rGO/C-ZIF-8 electrodes surface were observed via SEM analysis after 250 cycles in an LFP full cell, as shown in Figure 7a–c. After cycling, the surfaces of the Cu-Li and rGO-Li electrodes exhibited numerous cracks, whereas the rGO/C-ZIF-8 electrode surface remained relatively dense with fewer cracks even after 250 cycles. The presence of cracks accelerates electrolyte consumption, leading to decreased coulombic efficiency and poor cycling performance. This demonstrates that the rGO/C-ZIF-8 composite, with fewer cracks, can maintain stable cycling performance with minimal surface changes even after prolonged cycling.

## 4. Conclusions

We successfully synthesized rGO/C-ZIF-8 composites using a simple redox reaction and carbonization method, aimed at enhancing the stability of Li metal anodes. The lithiophilic Zn, N of C-ZIF-8, and the oxygen functional group of rGO acted as Li host sites to decrease the Li nucleation energy barrier for uniform Li deposition. The rGO/C-ZIF-8 exhibited stable Li plating/stripping performance at a current density of 1 mA cm^−^^2^ for an areal capacity of 1 mAh cm^−^^2^ for over 100 h. Notably, the electrode demonstrated low Li plating overpotential at high current densities (~72.6 mV at 5 mA cm^−^^2^, ~123.5 mV at 10 mA cm^−^^2^), highlighting its suitability for high-rate applications. Moreover, a full cell with a LiFePO_4_ cathode showed excellent cyclability, maintaining a capacity of 112 mAh g^−^^1^ (capacity retention 89%) for 250 cycles at a 1 C-rate, and exhibiting an excellent high-rate capability of 69.1 mAh g^−^^1^ at a 5 C-rate, further affirming the potential in practical applications. The design of the rGO/C-ZIF-8 composite, which integrates lithiophilic Zn and N with rGO, represents a promising approach to reduce overpotential and improve the overall performance of Li metal anodes. This strategy could significantly advance the development of dendrite-free Li metal batteries (LMBs), providing a viable path toward practical high-energy-density LMBs. Additionally, it indicates the feasibility of scaling up to LMBs while maintaining high energy density, a long cycle life, and enhanced safety.

## Data Availability

The original contributions presented in this study are included in this article. Further inquiries can be directed to the corresponding author.

## Supplementary Materials

The following supporting information can be downloaded at https://www.mdpi.com/article/10.3390/ma17174300/s1, Figure S1: Typical voltage profiles of Cu, rGO, and rGO/C-ZIF-8 during the initialization process; Figure S2: Li plating profiles of Cu, rGO, and rGO/C-ZIF-8; Figure S3: Charge/discharge profiles for different cycles of (a) LFP/Cu-Li and (b) LFP/rGO-Li; Figure S4: Charge/discharge profiles at different rates of (a) LFP/Cu-Li and (b) LFP/rGO-Li; Table S1: Nucleation energy barrier of Li on Cu, rGO, and rGO/C-ZIF-8 at various current densities with 1 mAh cm^−2^ areal capacity; Table S2: Failure time and plating/stripping overpotential of Li on Cu-Li, rGO-Li, and rGO/C-ZIF-8-Li at 1 mA cm^−2^ current densities with 1 mAh cm^−2^ areal capacity; Table S3: Plating/stripping overpotential of Li on Cu-Li, rGO-Li, and rGO/C-ZIF-8-Li at various current densities with 1 mAh cm^−2^ areal capacity.
