# Supplementary material for: Lithiophilic Reduced Graphene Oxide/Carbonized Zeolite Imidazolate Framework-8 Composite Host for Stable Li Metal Anodes
Source: Materials (Basel). 2024 Aug 30;17(17):4300. doi: 10.3390/ma17174300 (PMC11395789; doi:10.3390/ma17174300)
Supplement: Supplementary file 1 [file materials-17-04300-s001.zip › materials-3125867-SI.pdf]

Electronic Supplementary Information (ESI)

Lithiophilic Reduced Graphene Oxide/Carbonized ZIF-8 Composite host for Stable Li metal anode

Sang-Won Jeong[1, 2], Byeong Il Oh[1, 2], Eun Seo Chang[1, 2], Jeong-Ann Park[3, 4] *, and Hyun-Kyung Kim[1, 2] *

[1] Department of Battery Convergence Engineering, Kangwon University, 1, Kangwondaehak-gil, Chuncheon-si, Gangwon-do, Republic of Korea

[2] Interdisciplinary Program in Advanced Functional Materials and Devices Development, Kangwon National University, Chuncheon 24341, Republic of Korea

[3] Department of Environmental Engineering, Kangwon National University, Chuncheon, 24341, Republic of Korea

[4] Department of Integrated Energy and Infra System, Kangwon National University, Chuncheon, 24341, Republic of Korea

* Corresponding author: Prof. Hyun-Kyung Kim (hkk@kangwon.ac.kr) and Prof. Jeong-Ann Park (pjaan@kangwon.ac.kr)

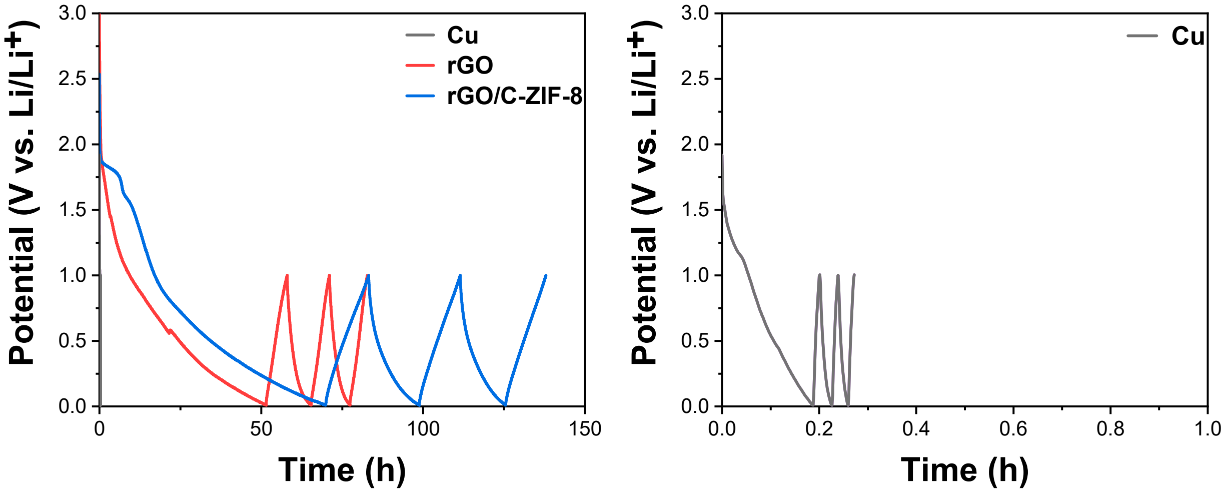

Figure S1. Typical voltage profiles of Cu, rGO, and rGO/C-ZIF-8 during initialization process.

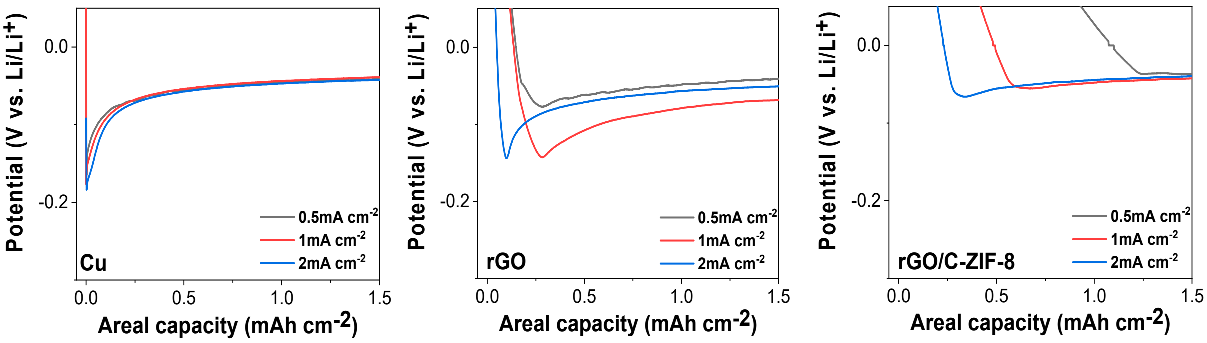

Figure S2. Li plating profiles of Cu, rGO, and rGO/C-ZIF-8

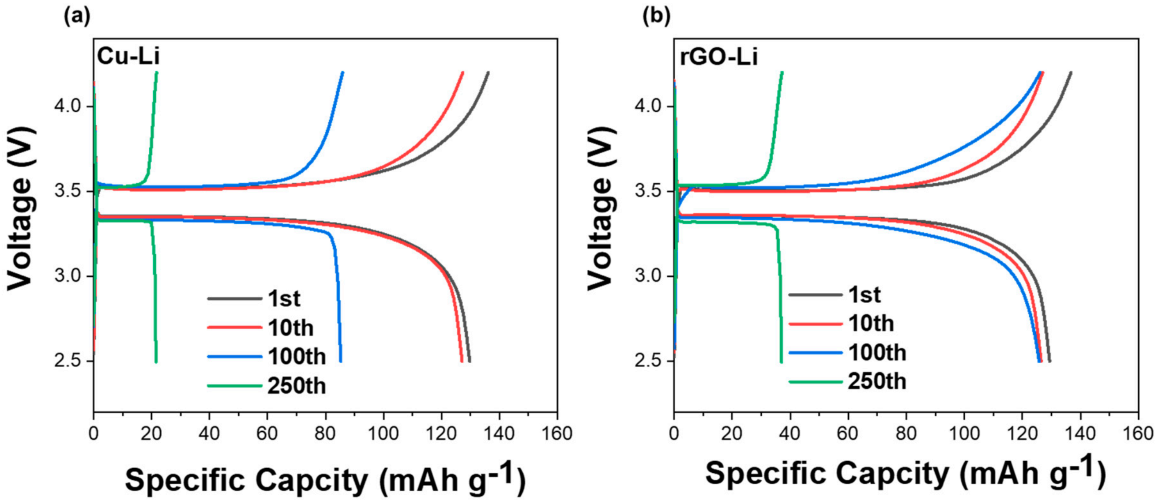

Figure S3. Charge/discharge profiles for different cycles of (a) LFP/Cu-Li and (b) LFP/rGO-Li.

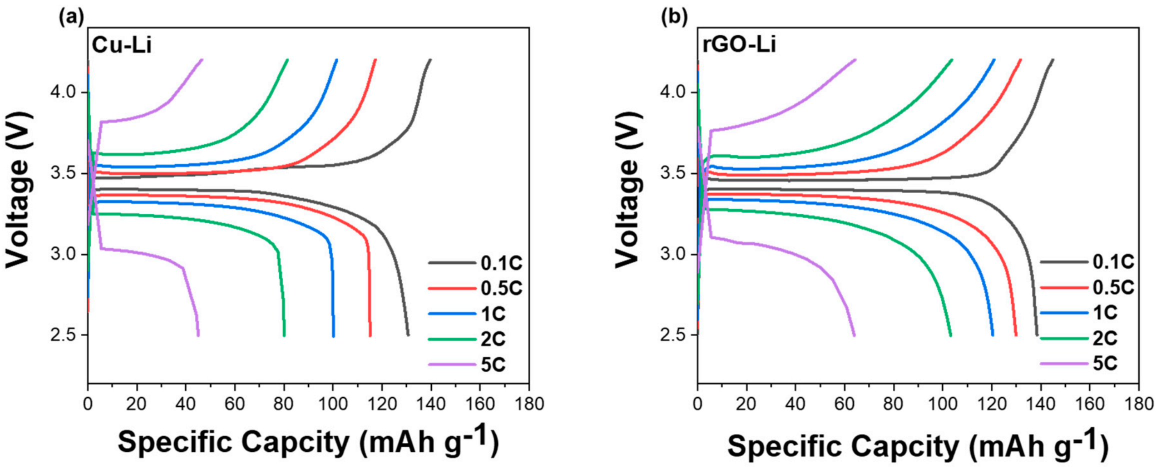

Figure S4. Charge/discharge profiles at different rates of (a) LFP/Cu-Li and (b) LFP/rGO-Li.

Table S1. Nucleation energy barrier of Li on Cu, rGO, and rGO/C-ZIF-8 at various current densities with 1mAh cm^{-2} areal capacity.

**Sample**	**Nucleation energy barrier**		
	**0.5 mA**	**1 mA**	**2 mA**
**Cu**	123.5 mV	132.7 mV	136.8 mV
**rGO**	36.7 mV	74.8 mV	93.4 mV
**rGO/C-ZIF-8**	16 mV	23.2 mV	29 mV

Table S2. Failure time and plating/stripping overpotential of Li on Cu-Li, rGO-Li, and rGO/C-ZIF-8-Li at 1mA cm^{-2} current densities with 1mAh cm^{-2} areal capacity.

Sample	Failure time	Li plating overpotential
**Cu-Li**	49 h	66.7 mV
**rGO-Li**	87 h	38.4 mV
**rGO/C-ZIF-8-Li**	-	30.5 mV

Table S3. Plating/Stripping overpotential of Li on Cu-Li, rGO-Li, and rGO/C-ZIF-8-Li at various current densities with 1mAh cm^{-2} areal capacity.

Sample	Li plating/stripping overpotential					
	**0.5mA cm^{-2}**	**1mA cm^{-2}**	**2mA cm^{-2}**	**3mA cm^{-2}**	**5mA cm^{-2}**	**10mA cm^{-2}**
**Cu-Li**	35.0 mV	51.2 mV	86.9 mV	162.2 mV	370.3 mV	224.5 mV
**rGO-Li**	21.6 mV	29.9 mV	41.2 mV	59.3 mV	90.2 mV	203.4 mV
**rGO/C-ZIF-8-Li**	21.2 mV	27.5 mV	40.8 mV	52.1 mV	72.6 mV	123.5 mV
